# Influence of O_2_ on the Erosion-Corrosion Performance of 3Cr Steels in CO_2_ Containing Environment

**DOI:** 10.3390/ma13030791

**Published:** 2020-02-09

**Authors:** Lei Xia, Yan Li, Leilei Ma, Hongmei Zhang, Na Li, Zhengyi Jiang

**Affiliations:** 1 School of Material and Metallurgy, University of Science and Technology Liaoning, Anshan 114051, China; xialei0627@163.com (L.X.); lilyzhm68@163.com (H.Z.); huatsing2006@163.com (N.L.); 2 State Key Laboratory of Metal Material for Marine Equipment and Application, Anshan 114009, China; 3 Iron & Steel Research Institutes of Ansteel Group Corporation, Anshan 114009, China; 4 Department of Information Engineering, Hebei GEO University, Shijiazhuang 050031, China; leilma@126.com; 5 School of Mechanical, Materials, Mechatronic and Biomedical Engineering, University of Wollongong, Wollongong, NSW 2522, Australia

**Keywords:** erosion-corrosion, 3Cr steel, CO_2_, O_2_

## Abstract

With the introduction of O_2_ during oil and gas production, the erosion-corrosion rate of tubing steels increases; the objective of this report is to explore the reason for this. Erosion–corrosion experiments were performed in environments of CO_2_ and CO_2_–O_2_, respectively. Macrographs, microstructures, and the compositions of erosion-corrosion scales were investigated using a digital camera, scanning electron microscope (SEM), Kevex-SuperDry energy spectrometer (EDS) and X-ray diffraction (XRD). The results show that the erosion-corrosion products are composed of large FeCO_3_ particles and some amorphous product in the CO_2_ environment, while they are made up of FeCO_3_, Fe_2_O_3_, Fe_3_O_4_, and bits of amorphous product in the CO_2_–O_2_ environment. The interface between erosion-corrosion scales and the substrate of 3Cr steel is smooth, and Cr enrichment obviously exists in the erosion-corrosion products in the CO_2_ condition. However, the erosion-corrosion scale is loose and porous with little Cr enrichment in the CO_2_–O_2_ environment, which makes the protectiveness of the erosion–corrosion scale weak, and pitting corrosion occurs. The addition of O_2_ may destroy the protective FeCO_3_ scale and Cr enrichment in the erosion-corrosion scale, which may be the main reason for the decline in the level of protectiveness of the erosion-corrosion scale, making it weak in terms of preventing the corrosive medium from diffusing to the substrate.

## 1. Introduction

In the later stages of oilfield development, the addition of an oil displacement agent into the oil well is a common technology used to increase oil recovery [1]. Carbon dioxide (CO_2_) has been studied for many years as a widely-used oil displacement agent [2]. It can expand the volume and reduce the viscosity of crude oil [3]. Much equipment employed in the production of oil and gas is made up of steel, such as pipelines. Dry CO_2_ does not corrode steel. However, there is formation water in oil and gas extraction, and this can significantly promote electrochemical corrosion in steel in humid or water-containing environments, leading to uniform and/or local corrosion [4].

CO_2_ corrosion is an important degradation mechanism for tubing in the oil and gas industries. It could shorten the service life of pipelines and affect normal production [5,6,7,8,9]. In flow systems, erosion occurs between corrosive media and pipeline materials due to their relative motion. Corrosion and erosion will affect each other, resulting in faster failure of the pipeline. A certain amount of alloying elements can be doped in the steel to enhance erosion-corrosion resistance. The first choice should be elements with good corrosion resistance and low price. Steel doped with chromium (Cr) is frequently employed to significantly increase the anti-CO_2_ corrosion ability of the tubing material and decrease the probability of local corrosion and/or pitting. The service time of the material can be significantly extended by increasing the concentration of Cr. When the concentration of Cr reaches 3%, the corrosion rate decreases significantly [10]. Therefore, 3Cr steel has been widely used as pipeline steel.

O_2_ contamination will inevitably occur during oil and gas production by adding chemicals, shutdowns of newly-installed piping, leaky fittings, and oil recovery [11,12]. In addition, in the process of thickened oil thermal extraction, a high-temperature fluid which contains a great deal of oxygen (O_2_) as well as water (H_2_O), CO_2_, and nitrogen (N_2_) is injected into oil wells [13]. O_2_ coexisting with CO_2_ can make environmental corrosiveness more severe and cause an increase in the erosion-corrosion rate of tubing steels [14]. Great efforts have been made to minimize CO_2_ corrosion [5,6,7,8,9], but research on the erosion-corrosion performance of tubing steels under a CO_2_–O_2_ condition is far from adequate, especially in terms of erosion-corrosion performance comparisons between aqueous CO_2_ systems with and without O_2_. In this paper, the erosion-corrosion performance of 3Cr steel in a CO_2_–O_2_ environment was investigated in comparison with that of a CO_2_ environment. The erosion-corrosion rates were obtained by observing weight loss. Macrographs of the erosion-corrosion scale were obtained using a digital camera. The microstructures and compositions of the erosion-corrosion scale were analyzed by scanning electron microscopy (SEM), energy spectrometer (EDS), and X-ray diffraction (XRD).

## 2. Materials and Methods 

**Figure 1:**
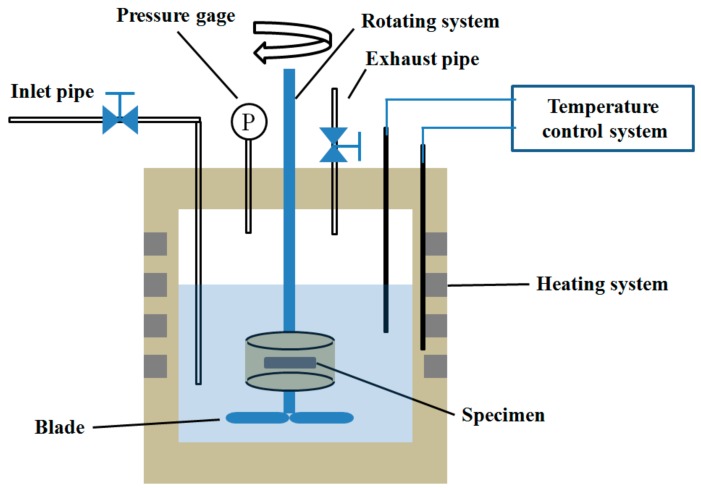
Schematic diagram of the HTHPE.

**Figure 2:**
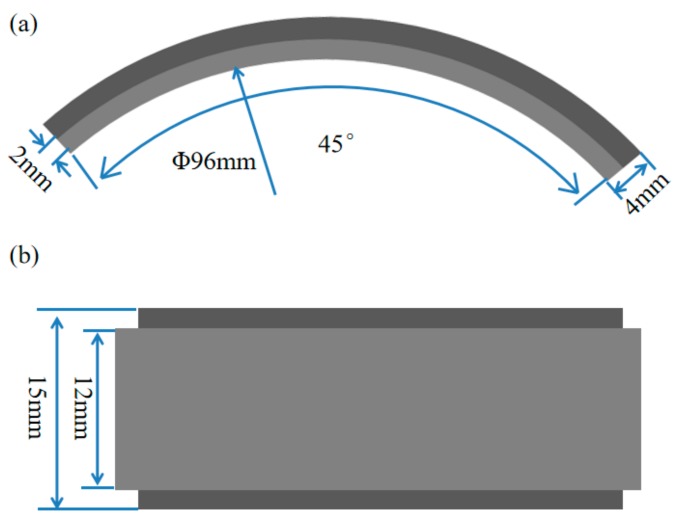
The shape and size of specimen (**a**) side view; (**b**) top view.

The erosion-corrosion test was conducted using high-temperature and high-pressure equipment (HTHPE) in environments of CO_2_ and CO_2_–O_2_, respectively. Figure 1 displays a schematic diagram of the HTHPE. It is principally composed of a gas control system, a heating system, a temperature control system, and a rotating system. The gas control system comprises an inlet pipe, an exhaust pipe, and a pressure gage. CO_2_ and/or O_2_ can enter the HTHPE through the air inlet pipe, and they can be discharged from the HTHPE through the exhaust pipe. The pressure of CO_2_ and/or O_2_ in the HTHPE is controlled by the pressure gage. The heating system can heat the HTHPE to elevate the system temperature; the temperature is controlled by a temperature control system. There is a rotating rod in the rotating system, and there are two stirring blades at the bottom of the rotating rod to stir the solution in the HTHPE. Specimens can be fixed on the rotating rod and rotate with it. The shape and size of the specimens are shown in Figure 2; they comprised a 45° stepped circular arc with an inner diameter of 96 mm and a thickness of 4 mm. The width of the outer ring was 15 mm and that of the inner ring was 12 mm.

**Figure 3:**
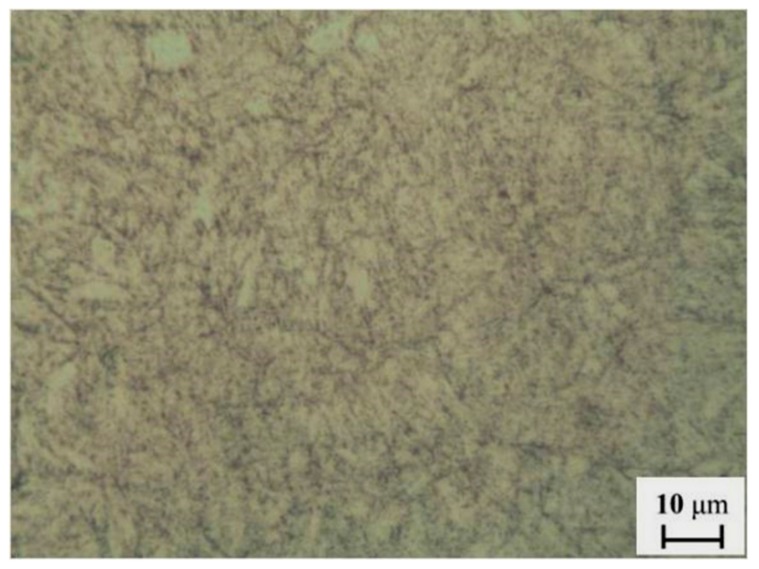
Microstructure of 3Cr steel.

The specimens were made of 3Cr steel. The chemical compositions of the tested material are as follows: C 0.37 at.%, Si 0.48 at.%, Mn 0.51 at.%, S < 0.009 at.%, P < 0.02 at.%, Cr 3.37 at.%, Ni 0.03 at.%, Ti 0.01 at.%; the rest is Fe. Figure 3 shows the microstructure of the specimen, which reveals acicular ferrite. Before the experiments, the working surface of the specimens was ground with silicon carbide paper to 800 grit. The specimens were cleaned with an ultrasonic cleaning device (GA008, Shenzhen Guanboshi industrial technology co. LTD, Shenzhen, China) for 20 min in acetone to remove grease, and then cleaned with alcohol and dried. They were weighed using an electronic analytical balance with an accuracy of 0.1 mg. The surfaces of the specimens, except for the working surface, were coated with 704 silicone rubber (Guangdong Hengda new material technology co. LTD, Huizhou, China), so that they would be isolated from the corrosive environment. The specimens were then fixed onto a rotating rod. Three of them are employed to study the average erosion-corrosion rate. The others were used for analyses of the structure and composition of the erosion-corrosion products. A certain amount of simulated solution was added to the HTHPE to simulate the water conditions of an oil and gas field. The component concentration of the test solution is given in Table 1, which ignores oily compounds in water extracted from the oil and gas field. The specimens were submerged in the solution. The experiment conditions are given in Table 2. The flow velocity given in Table 2 means that the linear velocity of the sample rotation was used to approximate the relative velocity between the samples and the liquid.

After the erosion-corrosion experiment, the specimens were removed from the HTHPE. The specimens were washed with deionized water and analytically pure alcohol, and then dried. Macrographs of the erosion-corrosion scales and the metal substrate after removal of the erosion-corrosion scales were obtained using a digital camera. The erosion-corrosion products of three specimens were removed by chemical cleaning. The cleaning solution was made up of 500 mL deionized water, 500 mL HCl, and 3.5 g urotropine (C_6_H_12_N_4_). The specimens were weighed after wiping; the average erosion-corrosion rate was calculated using Equation (1):

(1) $$C_{R}=\frac{8.76\times 10^{4}\times \left(m_{0}-m_{1}\right)}{\mathrm{St}\rho }$$

where C_R_ is the average erosion-corrosion rate, mm/a; m_0_ is the weight of original specimen, g; m_1_ is the weight of the specimen after removing the erosion-corrosion product film, g; t is the erosion-corrosion time, h; ρ is the density of the specimen, g/cm^3^; and S is the erosion-corrosion surface area of specimens, cm^2^. 

The microstructure and elemental composition of the erosion-corrosion scales on the specimen surface under different conditions were analyzed using a SEM (ULTRA 55 SEM, Carl Zeiss AG, Jena, Germany) and EDS (INCA X-MAX 50 EDS, Oxford Instruments, Oxford, UK). The erosion-corrosion product powder was prepared by scraping the erosion-corrosion product off the surface of the specimens with a blade, and a semiquantitative analysis of the erosion-corrosion product film was carried out by XRD (D8 Advance XRD, Bruker Corporation, Karlsruhe, Germany) with the internal standard of ZnO [15]. The mass ratio of ZnO and erosion-corrosion products was 3:7.

## 3. Results and Discussion

**Figure 4:**
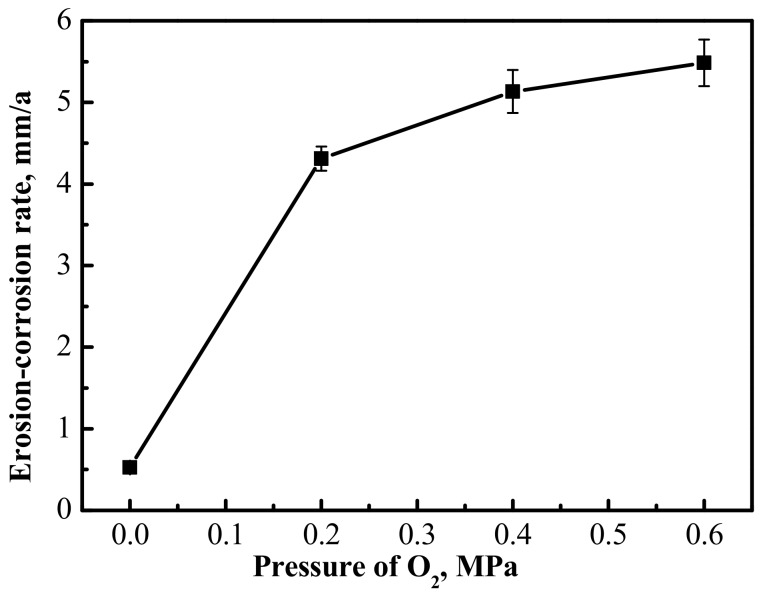
Average erosion-corrosion rate of 3Cr steel.

The average erosion-corrosion rate of the 3Cr steel was calculated after the erosion-corrosion experiments; the results are shown in Figure 4. It turns out from the data in Figure 4 that the erosion-corrosion rate increased as the O_2_ partial pressure went up. There was a significant difference in the erosion-corrosion rate of 3Cr steel comparing the CO_2_ and the CO_2_–O_2_ conditions. It was only about 0.5 mm/a in the CO_2_ environment, but increased significantly when there was 0.2 MPa O_2_ in the environment, reaching about 4.3 mm/a. Compared with the change from 0 MPa to 0.2 MPa in the partial pressure of O_2_, the erosion-corrosion rate of 3Cr steel increased slowly when the O_2_ partial pressure increased from 0.2 MPa to 0.6 MPa. 

**Figure 5:**
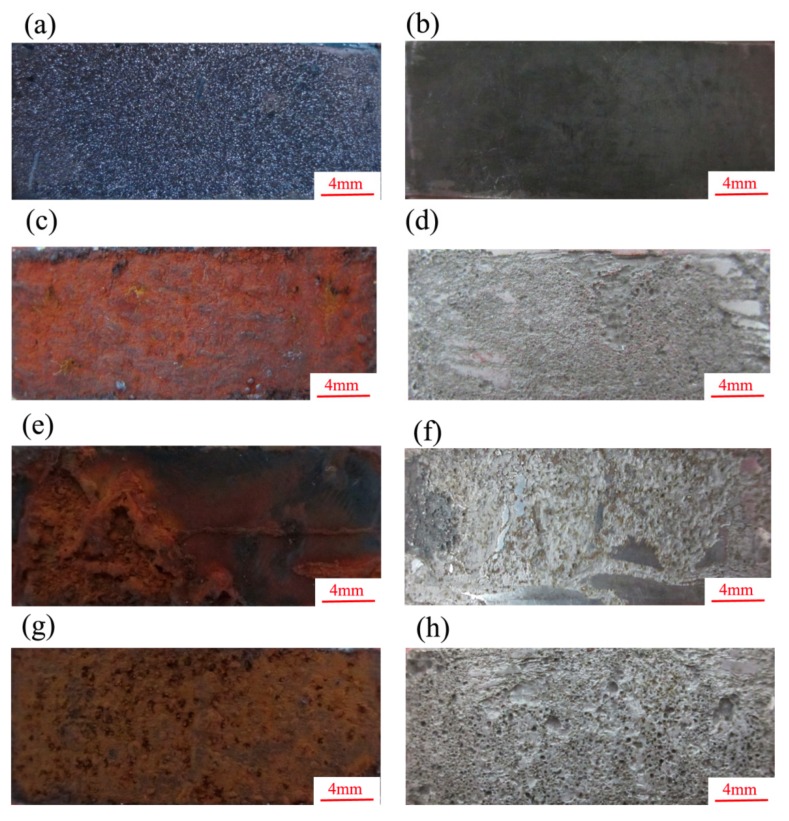
Macrostructure of erosion-corrosion products and substrates after removal erosion-corrosion products under different conditions (**a**) P_CO__2_: 2.5 MPa, erosion-corrosion product, (**b**) P_CO__2_: 2.5 MPa, substrate, (**c**) P_CO__2_: 2.5 MPa, P_O__2_: 0.2 MPa, erosion-corrosion product, (**d**) P_CO__2_: 2.5 MPa, P_O__2_: 0.2 MPa, substrate, (**e**) P_CO__2_: 2.5 MPa, P_O__2_: 0.4 MPa, erosion-corrosion product, (**f**) P_CO__2_: 2.5 MPa, P_O__2_: 0.4 MPa, substrate, (**g**) P_CO__2_: 2.5 MPa, P_O__2_: 0.6 MPa, erosion-corrosion product, (**h**) P_CO__2_: 2.5 MPa, P_O__2_: 0.6 MPa, substrate.

The macrostructure of the erosion-corrosion scales and the substrate after the removal of the erosion-corrosion products are presented in Figure 5. As shown, the erosion-corrosion products were dense, and the substrate was flat and smooth under the condition of CO_2_ (Figure 5a,b). In the CO_2_–O_2_ environment, the erosion-corrosion product was loose and reddish-brown (Figure 5c,e,g); the surface of the substrate after their removal was quite rough, with several pits of different sizes (Figure 5d,f,h). A certain amount of erosion-corrosion product fell off the surface of the specimens when the pressure of O_2_ was 0.6 MPa in the environment. 

**Figure 6:**
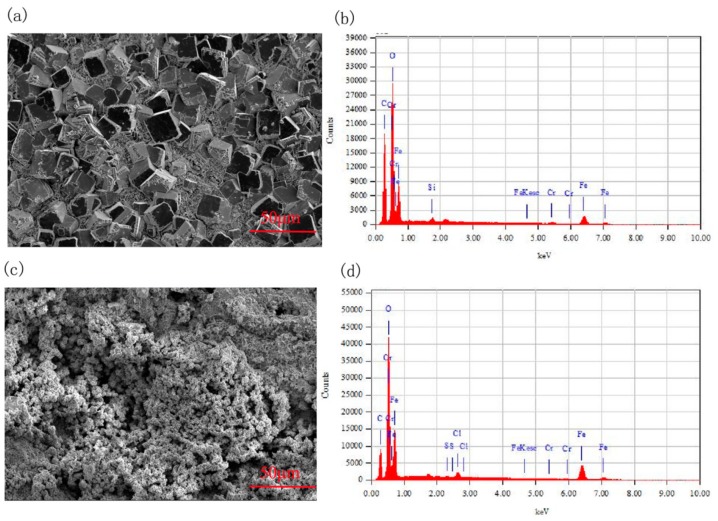
SEM and elemental analysis of erosion-corrosion products in CO_2_ and CO_2_–O_2_ environments (**a**) P_CO__2_: 2.5 MPa, surface morphology, (**b**) P_CO__2_: 2.5 MPa, elemental analysis, (**c**) P_CO__2_: 2.5 MPa, P_O__2_: 0.6 MPa, surface morphology, (d) P_CO__2_: 2.5 MPa, P_O__2_: 0.6 MPa, elemental analysis.

To further explore the microstructure and components of the erosion-corrosion products in CO_2_ and CO_2_–O_2_ environments, SEM and EDS analysis were performed. The micro image and elemental composition are set out in Figure 6 and Table 3. From Figure 6, we can see that grains of different sizes are stacked tightly on the sample surface in the CO_2_ environment, while there are no obvious grains on the surface of the specimen; the shape of the erosion-corrosion products is irregular in the CO_2_–O_2_ environment. The components of the erosion-corrosion scales were mainly elemental C, O, and Fe in the CO_2_ and CO_2_–O_2_ environments. The atomic ratio of elemental O and Fe was about 3 under the condition of CO_2_, while it was about 1.7 in the CO_2_–O_2_ environment. The elemental Cr content in the erosion-corrosion products was obviously lower than that in the substrate of 3Cr steel under the conditions of CO_2_ and CO_2_–O_2_. 

**Figure 7:**
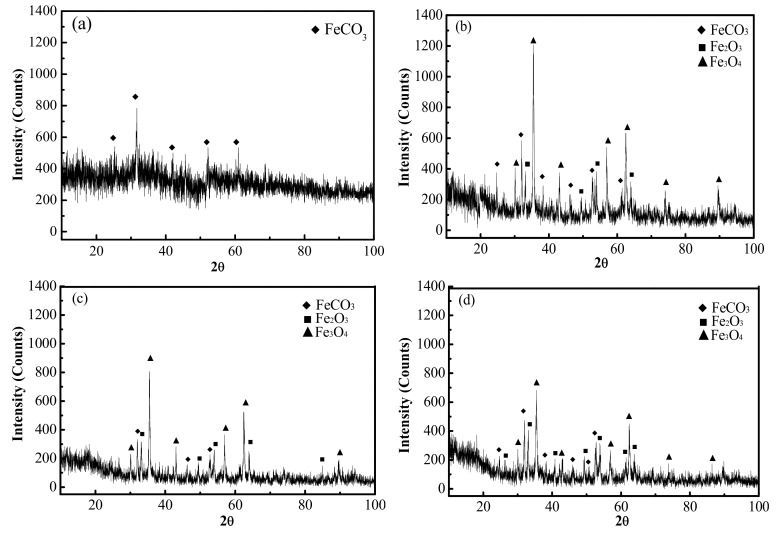
XRD analysis of erosion-corrosion products of 3Cr steel under different conditions (**a**) P_CO__2_: 2.5 MPa, (**b**) P_CO__2_: 2.5 MPa, P_O__2_: 0.2 MPa, (**c**) P_CO__2_: 2.5 MPa, P_O__2_: 0.4 MPa, (**d**) P_CO__2_: 2.5 MPa, P_O__2_: 0.6 MPa.

The composition of the erosion-corrosion products under different conditions was also analyzed by XRD, as set out in Figure 7. The data obtained from Figure 7 indicates that the erosion-corrosion scale was generally made up of FeCO_3_ in the CO_2_ environment, which is consistent with the atomic ratio of elemental O and Fe in Table 3. In addition to FeCO_3_, there were also Fe_2_O_3_ and Fe_3_O_4_ in the erosion-corrosion products under the condition of CO_2_–O_2_. There was also a certain amount of amorphous erosion-corrosion product in the CO_2_ and CO_2_–O_2_ environments. Kermani et al. demonstrated that doping steel with Cr causes the erosion-corrosion product film to transform from a crystalline to an amorphous state, the composition of which is mainly Cr(OH)_3_, Cr_2_O_3_ and/or some FeCO_3_ [16]. Comparing with the CO_2_ environment, there were fewer amorphous erosion-corrosion products in the CO_2_–O_2_ environment. A semiquantitative calculation of the crystalline erosion-corrosion scales of the tested steels is presented in Table 4. The data shows that the quantity of FeCO_3_ decreases, and the amount of Fe_2_O_3_ and Fe_3_O_4_ increases when the O_2_ pressure rises from 0 MPa to 0.4 MPa. However, there was some increase for the amount of FeCO_3_, while the amount of Fe_2_O_3_ and Fe_3_O_4_ decreased when the O_2_ pressure reached 0.6 MPa.

**Figure 8:**
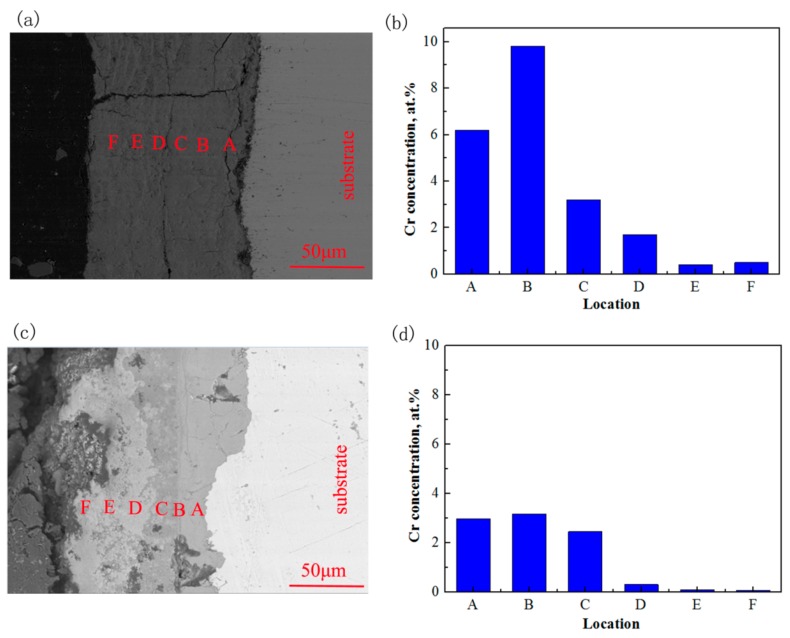
Cross-section morphologies and Cr elements concentration of erosion-corrosion product (**a**) P_CO__2_: 2.5 MPa, Cross-section morphology, (**b**) P_CO2_: 2.5 MPa, Cr element concentration, (**c**) P_CO2_: 2.5 MPa, P_O2_: 0.6 MPa, Cross-section morphology, (**d**) P_CO2_: 2.5 MPa, P_O2_: 0.6 MPa, Cr element concentration.

Figure 8 displays the cross-section morphologies and the concentration of Cr in the erosion-corrosion products under CO_2_ and CO_2_–O_2_ environments. As shown in Figure 8, the interface between the erosion-corrosion products and the substrate was flat in the CO_2_ environment. The concentration of Cr increases and then decreases gradually with the erosion-corrosion product from inside to outside. Comparing with the Cr element concentration in the steel substrate, it was obviously higher in the erosion-corrosion product near the substrate. The erosion-corrosion products may be divided into two distinct layers, and the interface between erosion-corrosion products and the substrate is relatively rough in the CO_2_–O_2_ environment. The concentration of Cr in the inner erosion-corrosion product was similar to that of the substrate. However, there was only a small amount of Cr in the outer erosion-corrosion product under the condition of CO_2_–O_2_. 

In the CO_2_ environment, CO_2_ can react with H_2_O to form H_2_CO_3_, and some H_2_CO_3_ will be further dissociated to H^+^, HCO_3_^-^, and CO_3_^2−^ in the solution (Equation (2)–(4)). Therefore, the solution is slightly acidic. A lot of micro-corrosion cells can be formed at the interface between the 3Cr steel and the solution. The H^+^ is oxidized to hydrogen (H_2_), and Fe and Cr are reduced to Fe^2+^ and Cr^3+^ (Equation (5)–(7)). The solution on the steel surface gradually becomes slightly alkaline as the reaction progresses. The OH^−^ can react with Cr^3+^ to form Cr(OH)_3_ near the 3Cr steel surface (Equation (9)). Because the solubility product (K_sp_) of Cr(OH)_3_ is quite small, i.e., 6.3 × 10^−31^, some Cr(OH)_3_ may further react to form Cr_2_O_3_, according to Equation (10). Fe^2+^ can react with CO_3_^2−^ to form FeCO_3_ (Equation (11)), and the FeCO_3_ will be deposited on the surface of metal to form an erosion-corrosion product film. However, the generation of FeCO_3_ occurs later than that of Cr(OH)_3_, because the K_sp_ of FeCO_3_ (3.2 × 10^−11^) is larger than that of Cr(OH)_3_. That may be the reason why there is Cr enrichment in the inner erosion-corrosion scale in Figure 8b. Cr(OH)_3_ and Cr_2_O_3_ are amorphous, so they are not recognized by XRD in Figure 7. The erosion-corrosion scale is dense and stable in terms of preventing the corrosive medium from reaching the surface of the substrate effectively [17]. Therefore, the erosion-corrosion rate of 3Cr steel is quite low under the condition of CO_2_ (Figure 4).

(2) $$H_{2}O+{\mathrm{CO}}_{2}\to H_{2}{\mathrm{CO}}_{3}$$

(3) $$H_{2}{\mathrm{CO}}_{3}\to H^{+}+{\mathrm{HCO}}_{3}^{-}$$

(4) $${\mathrm{HCO}}_{3}^{-}\to H^{+}+{\mathrm{CO}}_{3}^{2-}$$

(5) $$2H^{+}+2e\to H_{2}$$

(6) $$\mathrm{Fe}-2e\to {\mathrm{Fe}}^{2+}$$

(7) $$\mathrm{Cr}-3e\to {\mathrm{Cr}}^{3+}$$

(8) $$H_{2}O\to H^{+}+{\mathrm{OH}}^{-}$$

(9) $${\mathrm{Cr}}^{3+}+3{\mathrm{OH}}^{-}\to \mathrm{Cr}{\left(\mathrm{OH}\right)}_{3}$$

(10) $$2\mathrm{Cr}{\left(\mathrm{OH}\right)}_{3}\to {\mathrm{Cr}}_{2}O_{3}+3H_{2}O$$

(11) $${\mathrm{Fe}}^{2+}+{\mathrm{CO}}_{3}^{2-}\to {\mathrm{FeCO}}_{3}$$

O_2_ may react with H_2_O and form OH^-^ near the 3Cr steel surface (Equation (12)). The solution will become alkaline. Fe^2+^ can be oxidized to Fe^3+^ by O_2_ (Equation (13)). Fe^2+^ and Fe^3+^ react with OH^-^ to form Fe(OH)_2_ and Fe(OH)_3_ (Equation (14), (15)). Some Fe(OH)_2_ will be further oxidized to Fe(OH)_3_ by O_2_ (Equation (16)). Fe_2_O_3_ and Fe_3_O_4_ can be formed through Equation (17) and (18). Some FeCO_3_ may be oxidized to Fe_2_O_3_ according to Equation (19). Cr(OH)_3_, Fe_2_O_3_ and/or Fe_3_O_4_ may deposit on the surface of 3Cr steel simultaneously, which is not conducive to the formation of a dense and protective erosion-corrosion scale film. The erosion-corrosion scale will be loose and porous, so that a corrosive medium cannot be prevented from reaching the substrate of the steel under the condition of CO_2_–O_2_. Thus, the erosion-corrosion rate increases significantly when O_2_ is present in the environment (Figure 4). There will be more dissolved O_2_ in the solution and at the interface of 3Cr steel and the solution, with the partial pressure of O_2_ increasing. This is conducive to the formation of Fe_2_O_3_ and/or Fe_3_O_4_ according to Equation (12)–(19) (Table 4), which will accelerate the erosion-corrosion of 3Cr steel. The outer erosion-corrosion product is mainly loose Fe_2_O_3_ and/or Fe_3_O_4_, which easily falls off the surface of 3Cr steel under the action of scouring. More Fe_2_O_3_ and/or Fe_3_O_4_ falls off the erosion-corrosion scales when the partial pressure of O_2_ reaches 0.6 MPa, resulting in a lower quantity of Fe_2_O_3_ and Fe_3_O_4_ (Table 4).

(12) $$2H_{2}O+O_{2}\to 4{\mathrm{OH}}^{-}$$

(13) $$4{\mathrm{Fe}}^{2+}+O_{2}+2H_{2}O\to 4{\mathrm{Fe}}^{3+}+4{\mathrm{OH}}^{-}$$

(14) $${\mathrm{Fe}}^{2+}+2{\mathrm{OH}}^{-}\to \mathrm{Fe}{\left(\mathrm{OH}\right)}_{2}$$

(15) $${\mathrm{Fe}}^{3+}+3{\mathrm{OH}}^{-}\to \mathrm{Fe}{\left(\mathrm{OH}\right)}_{3}$$

(16) $$4\mathrm{Fe}{\left(\mathrm{OH}\right)}_{2}+O_{2}+2H_{2}O\to 4\mathrm{Fe}{\left(\mathrm{OH}\right)}_{3}$$

(17) $$2\mathrm{Fe}{\left(\mathrm{OH}\right)}_{3}\to {\mathrm{Fe}}_{2}O_{3}+3H_{2}O$$

(18) $$2\mathrm{Fe}{\left(\mathrm{OH}\right)}_{3}+\mathrm{Fe}{\left(\mathrm{OH}\right)}_{2}\to {\mathrm{Fe}}_{3}O_{4}+4H_{2}O$$

(19) $$4{\mathrm{FeCO}}_{3}+O_{2}+4H_{2}O\to {\mathrm{Fe}}_{2}O_{3}+4H_{2}{\mathrm{CO}}_{3}$$

## 4. Conclusions

The erosion-corrosion rate of 3Cr steel apparent increases when there is O_2_ in the CO_2_ environment; this rate correlates with the partial pressure of O_2_.

The erosion-corrosion product is uniform with obvious Cr enrichment in a CO_2_ environment, composed of large FeCO_3_ particles and some amorphous erosion–corrosion products. However, the erosion-corrosion scale is loose and porous with little Cr enrichment in CO_2_–O_2_ environments. It is made up of FeCO_3_, Fe_2_O_3_, Fe_3_O_4_, and a bit of amorphous erosion-corrosion products. 

3Cr steel exhibits excellent erosion-corrosion performance under the condition of CO_2_. However, mixing with O_2_ may destroy the dense and protective FeCO_3_ and Cr enrichment in the erosion-corrosion product. The erosion-corrosion products become loose and porous, which is favorable for the corrosive medium to diffuse into the steel surface and the erosion-corrosion product. Thus, the erosion-corrosion rate increases significantly, and pitting corrosion may occur under the condition of CO_2_–O_2_.

## Figures and Tables

**Table 1:** Chemical composition of formation water drawn from the oil field.

Composition	Content (mg/L)
MgCl_2_.6H_2_O	73.8
CaCl_2_	49.9
Na_2_CO_3_	383.6
NaHCO_3_	1022.7
Na_2_SO_4_	7.1
NaCl	543.2
KCl	1.8

**Table 2:** Experimental parameters.

Test No.	Pressure of CO_2_ (MPa)	Pressure of O_2_ (MPa)	Temperature (°C)	Flow Velocity (m/s)	Time (Hour)
A	2.5	0	120	1	120
B	2.5	0.2	120	1	120
C	2.5	0.4	120	1	120
D	2.5	0.6	120	1	120

**Table 3:** Quantitative EDS results of erosion-corrosion products of the tested material under different conditions.

Conditions	Elements Concentration, at.%						
	C	O	S	Cl	Cr	Si	Fe
P_CO__2_: 2.5 MPa	50.23	37.79	-	-	0.65	0.29	11.04
P_CO__2_: 2.5 MPa P_O__2_: 0.6 MPa	28.54	43.96	0.10	0.90	0.13	-	26.36

**Table 4:** XRD semiquantitative calculation data of erosion-corrosion scales of tested steels in different conditions.

Tested Conditions	XRD Results, wt %		
	FeCO_3_	Fe_3_O_4_	Fe_2_O_3_
P_CO__2_: 2.5 MPa	100	--	--
P_CO__2_: 2.5 MPa, P_O__2_: 0.2 MPa	33.2	19.0	47.8
P_CO2_: 2.5 MPa, P_O2_: 0.4 MPa	24.0	26.2	49.8
P_CO2_: 2.5 MPa, P_O2_: 0.6 MPa	34.5	33.4	32.1
